# The reliability of three-dimensional scapular attitudes in healthy people and people with shoulder impingement syndrome

**DOI:** 10.1186/1471-2474-8-49

**Published:** 2007-06-21

**Authors:** Jean-Sébastien Roy, Hélène Moffet, Luc J Hébert, Guy St-Vincent, Bradford J McFadyen

**Affiliations:** 1 Centre for Interdisciplinary Research in Rehabilitation and Social Integration, Quebec City, Quebec, Canada; 2 Department of Rehabilitation, Faculty of Medicine, Laval University, Quebec City, Quebec, Canada; 3 Department of Radiology, Faculty of Medicine, Laval University, Quebec City, Quebec, Canada; 4 National Defence of Canada, CFHS Headquarters, Ottawa, Ontario, Canada

## Abstract

**Background:**

Abnormal scapular displacements during arm elevation have been observed in people with shoulder impingement syndrome. These abnormal scapular displacements were evaluated using different methods and instruments allowing a 3-dimensional representation of the scapular kinematics. The validity and the intrasession reliability have been shown for the majority of these methods for healthy people. However, the intersession reliability on healthy people and people with impaired shoulders is not well documented. This measurement property needs to be assessed before using such methods in longitudinal comparative studies. The objective of this study is to evaluate the intra and intersession reliability of 3-dimensional scapular attitudes measured at different arm positions in healthy people and to explore the same measurement properties in people with shoulder impingement syndrome using the Optotrak Probing System.

**Methods:**

Three-dimensional scapular attitudes were measured twice (test and retest interspaced by one week) on fifteen healthy subjects (mean age 37.3 years) and eight subjects with subacromial shoulder impingement syndrome (mean age 46.1 years) in three arm positions (arm at rest, 70° of humerothoracic flexion and 90° of humerothoracic abduction) using the Optotrak Probing System. Two different methods of calculation of 3-dimensional scapular attitudes were used: relative to the position of the scapula at rest and relative to the trunk. Intraclass correlation coefficient (ICC) and standard error of measure (SEM) were used to estimate intra and intersession reliability.

**Results:**

For both groups, the reliability of the three-dimensional scapular attitudes for elevation positions was very good during the same session (ICCs from 0.84 to 0.99; SEM from 0.6° to 1.9°) and good to very good between sessions (ICCs from 0.62 to 0.97; SEM from 1.2° to 4.2°) when using the method of calculation relative to the trunk. Higher levels of intersession reliability were found for the method of calculation relative to the trunk in anterior-posterior tilting at 70° of flexion compared to the method of calculation relative to the scapula at rest.

**Conclusion:**

The estimation of three-dimensional scapular attitudes using the method of calculation relative to the trunk is reproducible in the three arm positions evaluated and can be used to document the scapular behavior.

## Background

Recently, more attention has been given to characterize scapular mobility in shoulder dysfunctions [1,2]. Shoulder impairments, such as subacromial shoulder impingement syndrome (SIS), have been associated with abnormal movement of the scapula during elevation of the arm [1-6]. Some authors reported that, when compared with the non-impaired side or to healthy people, the shoulders with impingement have shown, during arm elevation, significantly less posterior tilting [2-4,6], as well as significant reduction of lateral rotation [3,4], and increased protraction of the scapula under a loading condition [4]. Others have hypothesized that people with less posterior tilting on the impaired side as compared to the controlateral shoulder may be at risk of developing chronic SIS [1]. These findings highlight the importance of considering scapular movements during both evaluation and treatment of the shoulder complex.

Scapular movements during elevation of the arm are the result of three scapular rotations. According to the International Society of Biomechanics (ISB) recommendations [7], the three scapular rotations are defined as: lateral/medial rotation (L-MR) (also called upward/downward rotation or external/internal rotation), anterior/posterior tilting (A-PT) (also called posterior/anterior tilting [8]), and protraction/retraction (PRO-RET) (also called external/internal rotation or anterior/posterior transverse rotation).

The scapula moves in a complex pattern during arm elevation [9-11]. The scapula has to move in large amplitudes around three different axes to optimize the length-tension relationship of the glenohumeral muscles and the position of the glenoid for an efficient shoulder movement during elevation of the arm [11,12]. This adds degrees of freedom to the shoulder and makes motion analysis of the scapula very challenging. The normal range of scapular movements during arm elevation varies considerably from one study to the others [11]. Some factors suggested to explain this large variation include the existence of different movement strategies used in the healthy population, but also a lack of standardization in measurement methods and instruments. For instance, differences in Euler angle sequence of rotations, in the orientation of the local reference system and in the type of instruments were noted [8,9,11,13-16]. These differences across studies support the ISB recommendation to adopt standards for joint coordinate systems to allow a better comparison between studies [7].

To improve the diagnostic process and the clinical follow-up of people with SIS, measurements of scapular movements must have good metric properties. A method for the measurement of the three-dimensional scapular attitudes (3DSA) using the Optotrak probing system (Northern Digital Inc., Waterloo, Ontario, Canada) was previously developed in our laboratory [8]. Scapular attitude measurements calculated with this method were found accurate and showed a good concurrent validity with a mean difference between the Optotrak probing system method and fixed infrared markers of only 1.7° on an anatomical model (the markers are a measurement standard provided by Northern Digital that were attached on the model). However, the reliability of this method in normal and impaired shoulders still needs to be established. This is an essential step before using this measurement to characterize scapular behavior in cross sectional and longitudinal clinical studies. The main objective of this study was to evaluate the intrasession and intersession reliability of the 3DSA using a method previously developed at different arm elevation positions on healthy people and to explore the same measurement properties in people with SIS [8]. The secondary objective was to compare the level of reliability obtained when using two different methods of calculation.

## Methods

### Subjects selection

Fifteen healthy subjects (mean age 37.3 years; eight female, seven male) and eight subjects with primary subacromial SIS (mean age 46.1 years; seven female, one male) voluntarily participated in the study (Table 1). The healthy subjects had no history of rheumatoid, inflammatory, degenerative or neurological diseases, as well as any previous surgery, pain or movement limitation to the shoulders. All subjects with SIS were assessed by an experienced shoulder orthopedic surgeon. They were included if they had at least one positive finding in each of these three categories [1]: painful arc of movement during active shoulder flexion or abduction; positive Neer [17] or Kennedy-Hawkins [18] impingement test; pain on resisted isometric lateral rotation and abduction or Jobe test [19]. Exclusion criteria were: bilateral SIS; calcification and fractures (evaluated by X-rays); shoulder instability; rheumatoid, inflammatory, degenerative or neurological diseases; previous neck or shoulder surgery; neck pain, cervicobrachialgia or shoulder pain reproduced during neck movement; shoulder capsulitis. When a rotator cuff tear was suspected, an ultrasound examination of the rotator cuff was performed by a radiologist [20]. All the participants read and signed an informed consent form. This study was approved by the Ethics Committee of the Quebec Rehabilitation Institute.

**Table 1:** Subjects' characteristics at baseline

Variables	Healthy subjects (n = 15)			SIS subjects (n = 8)		
		
	Mean	SD	Range	Mean	SD	Range
Age (years)	37.3	13.2	25–62	46.1	11.3	29–60
Height (m)	1.7	0.1	1.6–1.9	1.7	0.1	1.5–1.8
Weight (kg)	71.1	13.4	54.5–93.4	75.5	14.9	57.2–96.2
Duration of shoulder pain (months)				15.1	15.4	3–48
SPADI score session 1				53.4	15.0	34.6–79.9
SPADI score session 2				49.5	20.1	32.2–84.4
Sex	7 Men, 8 Women			1 Man, 7 Women		
Hand dominance	15 Right			5 Right, 3 Left		

### Study design

A test-retest design was used. Participants were involved in two measurement sessions one week apart (mean 6.3 ± 0.9 days) with the same protocol repeated by the same evaluator. The evaluator was not blinded to group status. However, he was blinded to the past values obtained in the first session when retesting during the second session. At each session, both shoulders were evaluated in three static positions: arm at rest, shoulder at 70° of humerothoracic flexion (in the sagittal plane), and shoulder at 90° of humerothoracic abduction (in the frontal plane). For the three arm positions, the glenohumeral joint was in a neutral position of axial rotation. The specific elevation positions were chosen because it has been shown that a reduced posterior tilting at 70° of flexion and 90° of abduction, along with five other variables, could explain as much as 91% of the variance of the pain and disability level experienced by people with SIS [21]. The study had two phases. First, the healthy subjects were evaluated. Their results were analyzed prior to the evaluation of the subjects with SIS in order to determine the influence of both the number of trials and the calculation methods of relative movements on 3DSA reliability. In the healthy group, three trials were recorded at each position and the level of intra and intersession reliability was compared using either the two first trials or all three recorded trials. Thereafter, the subjects with SIS were evaluated taking into account the findings in the healthy group. Only two trials in each position were recorded since the level of reliability was similar whether two or three trials were used (see results). To evaluate the stability of the shoulder condition between sessions, the subjects with SIS completed the Shoulder Pain And Disability Index (SPADI) at the beginning of each of the two measurement sessions [22].

### Measurements

**Figure 1:**
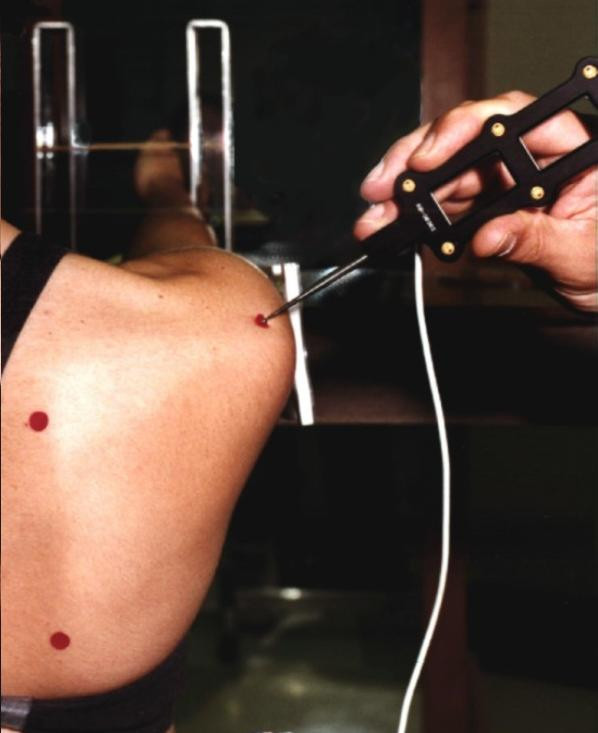
**Digitizing the scapular body landmarks**. Three non collinear landmarks on the scapula were digitized using the Optotrak probing accessory.

The 3DSA was calculated using the Optotrak Probing System (Northern Digital Inc., Waterloo, Ontario, Canada) with a standardized procedure [8]. For each trial, nine body landmarks were digitized using the Optotrak probing accessory (Figure 1). This probing accessory is attached to a rectangular rigid body incorporating six infrared transmitters used to define the coordinates of the tip of the probe, and therefore the coordinates of any point in contact with it [8]. The landmarks probed by the evaluator were, in this order: the acromial angle (most laterodorsal point of the scapula posterolateral tip of the acromion), the inferior angle (most caudal point of the scapula tip of the inferior angle) and the root of the spine (the midpoint of the triangular surface on the medial border of the scapula in line with the scapular spine), the medial and lateral epicondyles of the humerus (the most caudal point), the mid-upper arm (insertion of the deltoid), the C7 spinous process and the right and left posterosuperior iliac spines.

For the elevation positions, small stickers were placed on the skin nearby the location of the bony landmarks. Within a session, different stickers were used for each elevation position. The stickers were kept in place for the trials in the corresponding elevation position to allow the examiner to quickly locate each body landmark. The probe was then gently pressed against the skin at the identified location until a contact with the bone surface was felt. For all subjects, the landmarks were first probed with the arm at rest, for one trial, and then for the trials with the shoulder at 70° of flexion. Thereafter, the trial(s) with the arm at rest was(were) completed, followed by the trials with the shoulder at 90° of abduction.

Flexible templates, made of two plastic arms, were also used to locate and verify the position of the bony landmarks on the scapula across positions. A template was calibrated for each shoulder at the beginning of the first session. The apex of the template was located at the tip of the inferior angle of the scapula. One arm of the template was aligned from the apex to the posterolateral tip of the acromion, and the other arm from the apex to the medial inferior edge of the spine of the scapula. Before the first trial in each position, the position of the three landmarks on the scapula (marked by a sticker) was verified using the calibrated template. The same calibrated template was used for the second session of the same subject.

Subjects sat with their knees and hips flexed at 90° and their feet flat on the floor. A fluid goniometer (MIE Medical Research Ltd, Leeds, United Kingdom) was fixed to the upper arm to measure and verify the elevation angle. A table, placed next to the subject, was used to place the subject's arm 5° below the targeted elevation angle to allow the subjects to rest between the trials without going back to the resting position. During the task, the subjects simply had to unload their arm from the table and actively maintain the elevation angle while the bony landmarks were digitized. For the resting position, the subject was seated with their arms hanging to their side in a relaxed position. To ensure the subject had a stable posture and the same body position between trials and sessions, two supports were used, one resting against the thorax and another one fixed at the base of the skull. These supports were mounted on rigid adjustable bars fixed to a chair to minimize anterior-posterior movements of the trunk and the head.

**Figure 2:**
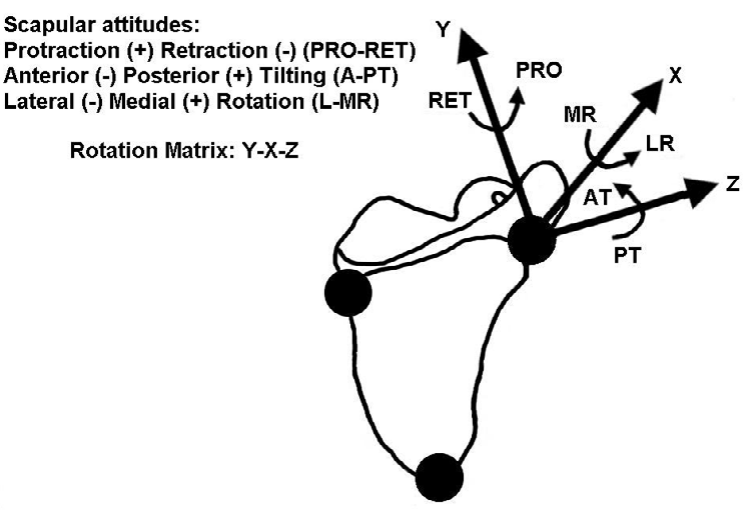
**Representation of the scapular rotations around the Y, X and Z axes**. The scapular rotations are defined in accordance with the ISB recommendations. The sequence of rotations used is YsXsZs.

The scapula coordinate system, the scapular reference frame and the Euler angle sequence of rotations (Y-X-Z order) were defined in accordance with the ISB recommendations [7] (Figure 2). The coordinate system (XsYsZs) had its origin coincident with the acromial angle. Zs was defined as the line connecting the root of the spine and the acromial angle, pointing to the acromial angle; Xs as the line perpendicular to the plane formed by the inferior angle, the acromial angle, and the root of the spine, pointing forward; and, Ys, as the common line perpendicular to the Xs- and Zs-axis, pointing upward [7].

Two methods of calculation of relative movements were used to determine the 3DSA of the healthy subjects. First, the position of the scapula with the arm in elevation was calculated relative to the position of the scapula with the arm at rest (the position of the scapula with the arm at rest was calculated using the global reference system). Second, the position of the scapula was calculated relative to the trunk. Since the method of calculation relative to the trunk was shown to be the most reliable for healthy subjects (see Results), it was the only one used for subjects with SIS.

### Data analysis

For all analyses of the healthy subjects, the results of the left and right shoulders were considered as independent observations, which led to the measurement of 30 shoulders of healthy subjects. The impaired shoulders (n = 8) and the non-impaired shoulder (n = 8) of the subjects with SIS were, however, independently analyzed. For the healthy subjects, the intrasession (intertrial) reliability was firstly analyzed by comparing the three trials in each position and then, by comparing the two first trials. For the intersession reliability, the mean of the three trials of the first session were compared to the mean of the three trials of the second session. The same intersession comparison was done for the means of the respective two first trials. For the subjects with SIS, the analyses for the intrasession reliability were carried out using the two trials that were performed, while the mean of the two trials for each session was used to assess the intersession reliability. The intra and intersession reliability was estimated by calculating the intraclass correlation coefficients (ICCs) and its 95% confidence interval (95%CI) (using SPSS 12.0; Reliability; Intraclass correlation coefficient) [23,24]. ICCs values were considered to reflect: a poor reliability when below 0.20; a fair reliability from 0.21 to 0.40; a moderatereliability from 0.41 to 0.60; a good reliability from 0.61 to 0.80 and, a very good reliability from 0.81 to 1.00 [25]. The standard errors of measurement (SEM) and its 95%CI were also calculated [26]. Significant differences in reliability between groups and between methods for the healthy subjects were determined when the 95% CI of the ICC and the SEM were not overlapping. Means and standard deviations of 3DSA in each scapular rotations and arm positions were calculated. Student *t*-tests were performed to verify if there were significant differences (p < 0.05) in SPADI scores between sessions.

## Results

**Figure 3:**
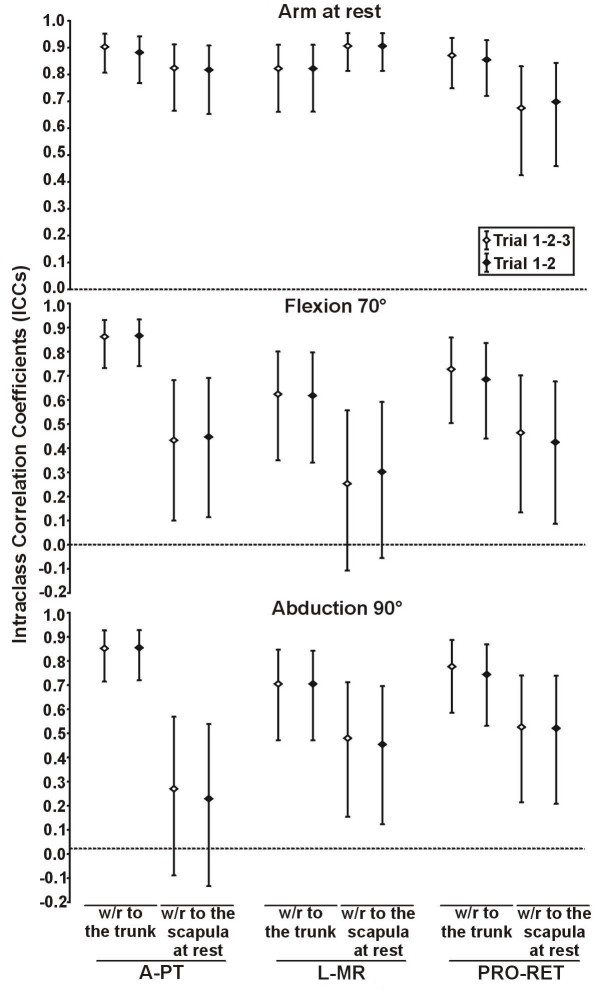
**Intersession intraclass correlation coefficients of 3D scapular attitudes using two methods of calculation**. The intersession intraclass correlation coefficients (ICCs) of 3D scapular attitudes (anterior/posterior tilting (A-PT), lateral/medial rotation (L-MR) and protraction/retraction (PRO-RET)) were measured in three static shoulder positions (arm at rest, 70° of flexion, 90° of abduction) using two methods of calculation (with respect (w/r) to the trunk and with respect (w/r) to the scapula at rest) in healthy subjects (n = 30 shoulders). The intersession ICCs were also measured using the mean of the three trials of each session (Trial 1-2-3) and the mean of the two first trials of each session (Trial 1–2). The error bar represents the 95% confidence interval of the ICCs.

**Figure 4:**
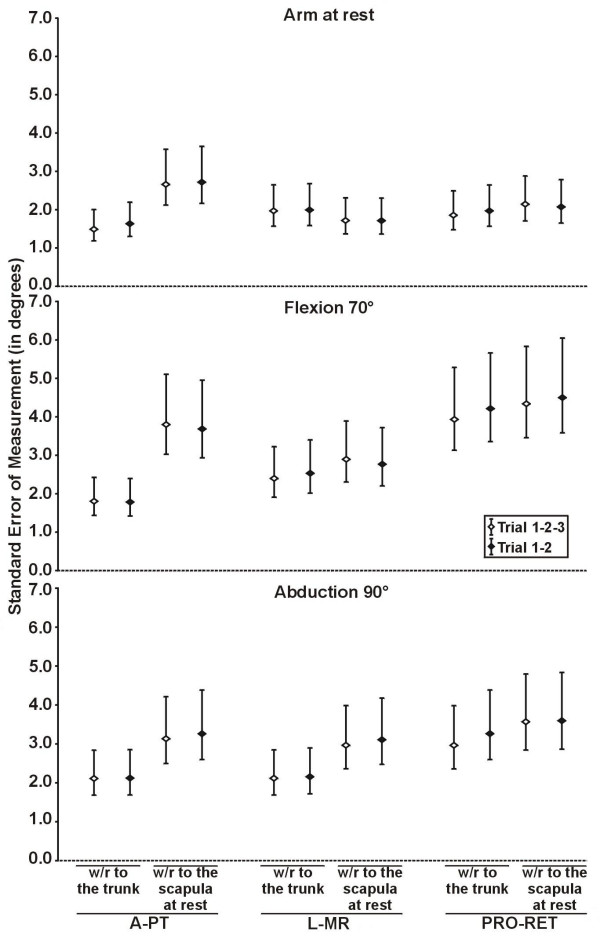
**Intersession standard error of measurement of 3D scapular attitudes using two methods of calculation**. The intersession standard error of measurement (SEM) of 3D scapular attitudes (anterior/posterior tilting (A-PT), lateral/medial rotation (L-MR) and protraction/retraction (PRO-RET)) were measured in three static shoulder positions (arm at rest, flexion, abduction) using two methods of calculation (with respect (w/r) to the trunk and with respect (w/r) to the scapula at rest) in healthy subjects (n = 30 shoulders). The intersession SEM were also measured using the mean of the three trials of each session (Trial 1-2-3) and the mean of the two first trials of each session (Trial 1–2). The error bar represents the 95% confidence interval of the SEM.

The level of reliability obtained when using two different methods of calculation of relative movement was assessed for the healthy subjects. Within the same session, reliability of both methods was good to very good (ICCs from 0.73 to 0.96 with 95%CI from 0.50 to 0.99 and SEM from 0.9° to 2.4° with 95%CI from 0.7° to 3.2°). Generally, the levels of between sessions reliability seemed better when using the method of calculation relative to the trunk (ICCs from 0.62 to 0.90 with 95%CI from 0.34 to 0.95 and SEM from 1.5° to 4.2° with 95%CI from 1.2° to 5.7° with the method relative to the trunk compared to ICCs from 0.23 to 0.91 with 95%CI from -0.13 to 0.95 and SEM from 1.7° to 4.5° with 95%CI from 1.4° to 6.0° with the method relative to the scapula at rest). However, the reliability was only significantly higher with the method of calculation relative to the trunk in A-PT at 70° of flexion for the ICCs and the SEM and in A-PT at 90° of abduction for the ICCs (Figure 3 and 4).

In healthy subjects, similar intra and intersession ICCs and SEM, with similar range for the 95%CI, were obtained using three trials and the two first trials, and no significant differences were found (Figures 3 and 4; Table 2). The mean 3DSA values (n = 2 trials, first session) and standard deviations calculated with the method relative to the trunk are shown in Table 4 for both groups.

**Table 2:** Intrasession standard errors of measurement of 3D scapular attitudes calculated in three static shoulder positions.

			Intrasession standard errors of measurement (in degrees)			
			
			Healthy subjects		SIS subjects	
					
					Impaired shoulder	Non-impaired shoulder
			n = 30 shoulders		n = 8 shoulders	n = 8 shoulders
				
Arm Positions	Scapular Rotations		Trials 1-2-3	Trials 1–2	Trials 1–2	Trials 1–2
Rest	A-PT	SEM	1.6	1.9	2.4	1.6
		95%CI	[1.4–2.0]	[1.5–2.5]	[1.6–4.8]	[1.1–3.2]
	L-MR	SEM	1.9	2.2	1.0	1.4
		95%CI	[1.6–2.3]	[1.8–3.0]	[0.7–2.0]	[1.0–2.9]
	PRO-RET	SEM	2.2	2.4	0.8	2.3
		95%CI	[1.9–2.7]	[1.9–3.2]	[0.5–1.5]	[1.5–4.7]
Flexion 70°	A-PT	SEM	1.0	1.2	1.1	0.6
		95%CI	[0.9–1.3]	[1.0–1.6]	[0.7–2.2]	[0.4–1.3]
	L-MR	SEM	1.3	1.2	1.5	0.8
		95%CI	[1.1–1.6]	[0.9–1.6]	[1.0–3.0]	[0.5–1.5]
	PRO-RET	SEM	1.9	1.6	0.8	1.4
		95%CI	[1.6–2.3]	[1.3–2.1]	[0.5–1.6]	[0.9–2.9]
Abduction 90°	A-PT	SEM	1.0	0.9	0.8	1.1
		95%CI	[0.8–1.2]	[0.7–1.2]	[0.5–1.6]	[0.7–2.3]
	L-MR	SEM	1.5	1.6	1.2	1.0
		95%CI	[1.3–1.8]	[1.3–2.1]	[0.8–2.4]	[0.7–2.1]
	PRO-RET	SEM	1.9	1.7	1.3	1.7
		95%CI	[1.6–2.3]	[1.4–2.3]	[0.8–2.6]	[1.1–3.5]

Abbreviations: A-PT, anterior-posterior tilting; L-MR, lateral-medial rotation; PRO-RET, protraction-retraction; SIS, shoulder impingement syndrome; Trial 1-2-3, standard errors of measurement calculated using the three trials of the first session; Trial 1–2, standard errors of measurement calculated using the two first trials of the first session; SEM, standard errors of measurement; 95%CI; 95% confidence interval.

The method of calculation relative to the trunk was used to calculate the 3D scapular attitudes.

**Table 4:** Mean 3D scapular attitudes (degrees) and standard deviations (SD) calculated in three static shoulder positions.

		Three-dimensional scapular attitudes (in degrees)					
		
		Healthy subjects		SIS subjects			
				
				Impaired shoulder		Non-impaired shoulder	
Arm Positions	Scapular Rotations	n = 30 shoulders		n = 8 shoulders		n = 8 shoulders	
				
		Mean	SD	Mean	SD	Mean	SD
Rest	A(-) P(+) T	-9.2	4.3	-7.4	3.7	-6.6	4.2
	L(-) M(+) R	-1.1	4.7	-7.6	3.7	-5.3	4.0
	PRO(+) RET(-)	-31.8	5.6	-33.3	6.8	-32.1	5.5
Flexion 70°	A(-) P(+) T	-3.8	4.5	-3.2	3.9	-1.9	5.2
	L(-) M(+) R	-9.3	3.7	-14.5	4.5	-12.4	4.2
	PRO(+) RET(-)	-39.8	7.5	-40.7	8.2	-39.5	6.7
Abduction 90°	A(-) P(+) T	-1.6	5.6	-1.7	5.2	-1.4	7.1
	L(-) M(+) R	-27.5	4.2	-30.4	4.7	-31.6	9.0
	PRO(+) RET(-)	-19.1	6.1	-18.6	8.7	-20.1	6.6

Abbreviations: A-PT, anterior-posterior tilting; L-MR, lateral-medial rotation; PRO-RET, protraction-retraction; SIS, shoulder impingement syndrome. The 3D scapular attitudes were calculated from the mean of the two first trials from the first session. The method of calculation relative to the trunk was used to calculate the 3D scapular attitudes.

The intrasession reliability for both groups was very good for all scapular rotations at 70° of flexion and 90° of abduction. ICCs ranging from 0.87 to 0.99 (with 95%CI from 0.67 to 1.00), and SEM ranging from 0.6° to 1.9° (with 95%CI from 0.4° to 3.5°) were found (Table 2). The intrasession reliability with the arm at rest was good to very good (ICCs from 0.65 to 0.97 with 95%CI from 0.43 to 0.99, SEM from 0.8° to 2.4° with 95%CI from 0.5° to 4.8°).

**Figure 5:**
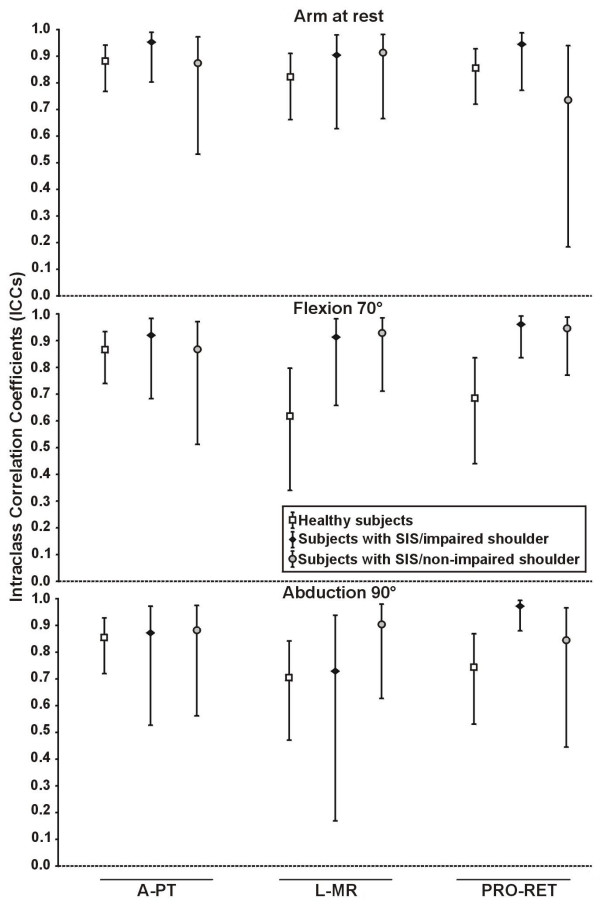
**Intersession intraclass correlation coefficients of 3D scapular attitudes for the two populations**. The intersession intraclass correlation coefficients (ICCs) of 3D scapular attitudes (anterior/posterior tilting (A-PT), lateral/medial rotation (L-MR) and protraction/retraction (PRO-RET)) was measured in three static shoulder positions (arm at rest, 70° of flexion, 90° of abduction) using the method of calculation relative to the trunk in healthy subjects (n = 30 shoulders) and subjects with SIS (n = 8 shoulders for impaired shoulders and non-impaired shoulders).

For the subjects with SIS, the intersession reliability for the arm at rest condition was good to very good for the three rotations (ICCs from 0.74 to 0.95 with 95%CI from 0.18 to 0.99, SEM from 0.7° to 2.8° with 95%CI from 0.5° to 5.6°). For the SIS shoulders alone (n = 8 shoulders), the reliability was good to very good in flexion and abduction (ICC from 0.73 to 0.97 with 95%CI from 0.17 to 0.99, SEM from 1.3° to 2.9° with 95%CI from 0.9° to 5.8°) (Figure 5 and Table 3). For the non-impaired shoulder alone, in flexion and abduction, the reliability was very good for all rotations (ICCs from 0.84 to 0.95 with 95%CI from 0.45 to 0.99, SEM from 1.2° to 2.7° with 95%CI from 0.8° to 5.6°) (Figure 5 and Table 3). Compared to the healthy subjects, significantly higher levels of reliability were found in the impaired shoulder of subjects with SIS in PRO-RET at 70° of flexion for the ICCs and the SEM and in PRO-RET at 90° of abduction for the ICCs. Significantly higher levels of reliability were also found in the non-impaired shoulder of subjects with SIS compared to healthy subjects for the SEM in L-MR with the arm at rest and in PRO-RET at 70° of flexion. There were no significant differences (p < 0.05) in the SPADI score between the two sessions (Table 1).

**Table 3:** Intersession standard errors of measurement of 3D scapular attitudes calculated in three static shoulder positions.

			Intersession standard errors of measurement (in degrees)		
			
			Healthy subjects	SIS subjects	
				
				Impaired shoulder	Non-impaired shoulder
Arm Positions	Scapular Rotations		n = 30 shoulders	n = 8 shoulders	n = 8 shoulders
Rest	A-PT	SEM	1.6	0.9	1.3
		95%CI	[1.3–2.2]	[0.6–1.7]	[0.8–2.6]
	L-MR	SEM	2.0	1.1	0.7
		95%CI	[1.6–2.7]	[0.7–2.2]	[0.5–1.5]
	PRO-RET	SEM	2.0	1.9	2.8
		95%CI	[1.6–2.6]	[1.3–3.9]	[1.8–5.6]
Flexion 70°	A-PT	SEM	1.8	1.3	1.8
		95%CI	[1.4–2.4]	[0.9–2.6]	[1.2–3.6]
	L-MR	SEM	2.5	1.3	1.2
		95%CI	[2.0–3.4]	[0.9–2.7]	[0.8–2.3]
	PRO-RET	SEM	4.2	1.6	1.6
		95%CI	[3.4–5.7]	[1.1–3.3]	[1.1–3.2]
Abduction 90°	A-PT	SEM	2.1	2.0	2.3
		95%CI	[1.7–2.9]	[1.3–4.0]	[1.5–4.6]
	L-MR	SEM	2.2	2.9	2.7
		95%CI	[1.7–2.9]	[1.9–5.8]	[1.8–5.6]
	PRO-RET	SEM	3.3	1.5	2.3
		95%CI	[2.6–4.4]	[1.0–3.1]	[1.5–4.7]

Abbreviations: A-PT, anterior-posterior tilting; L-MR, lateral-medial rotation; PRO-RET, protraction-retraction; SIS, shoulder impingement syndrome; SEM, standard errors of measurement; 95%CI; 95% confidence interval.

The method of calculation relative to the trunk was used to calculate the 3D scapular attitudes. The SEM were calculated using the mean of the two first trials of each session.

## Discussion

The 3DSA measurement method used in the present study has previously been shown to be accurate and valid [8]. The present results confirm its reliability for the measurement of 3DSA at rest and at two specific arm elevation angles, 70° of flexion and 90° of abduction, in healthy people and people with SIS. However, for some intersession reliability, the lower bound of the 95%CI of the ICCs were as low as 0.34 for the healthy subjects and 0.17 for subjects with SIS.

Other studies have evaluated the reliability of 3D scapular movements using different devices and procedures. Johnson et al. were one of the first groups to evaluate the repeatability of their 3D scapular movements method in abduction using the Isotrak electromagnetic system [15]. They reported a 95% confidence interval varying from 0.89° to 2.69° for the intraobserver (intrasession) variation. Meskers et al., using an electromagnetic device, reported standard deviations of 1.96° to 2.46° for intrasession variability and 3.03° to 4.17° for intersession variability for movements in flexion and abduction [13]. For movements in the plane of the scapula, other authors using also an electromagnetic device reported a very good intrasession reliability with ICCs above 0.90 and SEM ranging from 1.0° to 2.6° [14,16,27]. Cole et al. [6], using a 3D digitizer, reported intrasession ICC values above 0.80 in elevation in the plane of the scapula. Wang et al. [28] obtained ICCs above 0.85, except for PRO-RET with ICCs of 0.60, for intersession reliability with the arm at rest using a 3-D digitizer. The reliability of our measurement method (relative to the trunk) is as good as the ones reported in previous studies, showing small measurement errors within the same session and larger errors for intersession measurements. This is the first study to demonstrate the reliability of 3DSA with people who have a SIS.

The higher level of reliability in PRO-RET observed in the impaired shoulder of the subjects with SIS compared to healthy subjects could be explained by some group differences. Age and gender were different between the groups; the subjects with SIS were older and the proportion of men was lower (13% of men compared to 47% in the healthy group). A more restricted choice in motor strategies may also explain the higher level of reliability in PRO-RET. A study using functional magnetic resonance imaging have shown that the acromiohumeral distance in people with SIS varies very little and is smaller compared to healthy people around 90° of arm elevation [29]. It has been shown that people with SIS present changes in shoulder muscle activation during arm elevation, which may be associated with the reduction of the acromiohumeral distance [4,30-33]. These observations show that SIS shoulders may not perform the adequate movement strategy to avoid impingement and that persons with SIS seem to use the same repetitive movement pattern over time, which leaves a very small range of possible scapular motion. Finally, the number of subjects in the group with SIS was smaller. The estimation of the reliability uses different sources of variation of the measure [23,24]. These variations are more easily influenced by a small sample size.

Differences in scapular rotations in elevation positions between healthy people and those with SIS are relatively small [2,21]. Therefore, the error in the measurement of the 3DSA in elevation must be as small as possible. In the present study, two methods of calculation were used and compared in healthy subjects. For the method of calculation relative to the scapula at rest, the intersession reliability was fair to moderate at 70° of flexion and 90° of abduction with corresponding relatively low SEM. For the method relative to the trunk, the intersession reliability was good to very good with significantly higher ICCs in A-PT. With the calculation method relative to the scapula at rest, the reference position was obtained from a previous trial. For the method relative to the trunk, the reference position used is the one adopted by the subject during the same trial. Thus, an additional source of variation is introduced in the method relative to the scapula at rest. Our results, therefore, support the use of the 3DSA method of calculation relative to the trunk.

A reduced posterior tilting of the scapula during flexion or abduction was suggested as being a typical perturbation seen in people with SIS [2-4,6]. In a previous cross-sectional study [21], a small reduction of only 5° in posterior tilting of shoulders with SIS, as compared to contralateral healthy shoulders, has been related to a higher disability level. Small intersession SEM were found for this rotation both in healthy subjects (95%CI of 1.4 to 2.4° at 70° of flexion and 95%CI of 1.7 to 2.9° at 90° of abduction) and subjects with SIS (95%CI of 0.9° and 2.6° at 70° of flexion and 95%CI of 1.3° to 4.0°at 90° of abduction for the impaired shoulder). This suggests that the clinically important difference is very close to the superior limit of the 95%CI for the intersession SEM, especially for the SIS subjects. These findings highlight the importance of analyzing the changes in 3DSA in light of the errors associated with the measure.

In the present study, three trials at each arm position were recorded in healthy subjects to evaluate the impact of the number of trials recorded on repeatability. Our results showed that the level of intra and intersession reliability was not better when either two or three trials were used to estimate mean performances. Consequently, using the mean of two trials is advantageous since it provides reliable data in a shorter recording time.

One of the limits of the current proposed method is that 3DSA were measured in static positions. Thus, this method does not allow one to characterize dynamic changes in scapular attitudes from one position to another one. Also, the present results cannot be generalized to other shoulder positions since the method has only been tested in three arm positions. In addition to the variation in individual performance, other factors may explain the measurement errors found in the study. These other factors are related to the evaluator, the instruments and the measurement technique, including locating the landmarks, measuring arm position in elevation and manipulating the probe. A limited number of subjects was included in the group with SIS. This could explain the large variations in the 95%CI obtained for some rotations in this group. Finally, the use of the left and right shoulders of the healthy subjects as independent observations for the reliability could have lowered the between shoulder variability, thus influencing the ICCs and the SEM.

## Conclusion

The proposed 3DSA measurement method was found reliable in healthy and SIS subjects and the magnitude of the measurement error was determined for the three arm positions assessed. This method may be used to characterize changes in 3DSA over time and to measure the effect of rehabilitation intervention in people with SIS. In future studies, it will be important to analyze the findings in light of the magnitude of measurement errors.

## Competing interests

The author(s) declare that they have no competing interests.

## Authors' contributions

JSR participated in the design of the study, carried out the acquisition, the analysis and the interpretation of data and drafted the manuscript.

HM participated in the design of the study, in the analysis and in the interpretation of data and has been involved in drafting the manuscript.

LJH participated in the design of the study, in the analysis and in the interpretation of data and has been involved in drafting the manuscript.

GSV participated in the acquisition and the analysis of data, and has been involved in drafting the manuscript.

BJM participated in the design of the study, in the analysis and in the interpretation of data and has been involved in drafting the manuscript.

All authors read and approved the final manuscript.

## Pre-publication history

The pre-publication history for this paper can be accessed here:


